# Black metal thin films by deposition on dielectric antireflective moth-eye nanostructures

**DOI:** 10.1038/srep10563

**Published:** 2015-06-02

**Authors:** Alexander B. Christiansen, Gideon P. Caringal, Jeppe S. Clausen, Meir Grajower, Hesham Taha, Uriel Levy, N. Asger Mortensen, Anders Kristensen

**Affiliations:** 1 Department of Micro and Nanotechnology, Technical University of Denmark, Oersteds Plads, Building 345B, DK-2800 Kgs. Lyngby, Denmark; 2 Department of Photonics Engineering, Technical University of Denmark, Oersteds Plads, Building 343 DK-2800 Kgs. Lyngby, Denmark; 3 Department of Applied Physics, The Benin School of Engineering and Computer Science, The Center for Nanoscience and Nanotechnology, The Hebrew University of Jerusalem, Jerusalem 91904, Israel; 4 Nanonics Imaging Ltd., Hartum 19, Har Hotzvim, Jerusalem 97775, Israel

## Abstract

Although metals are commonly shiny and highly reflective, we here show that thin metal films appear black when deposited on a dielectric with antireflective moth-eye nanostructures. The nanostructures were tapered and close-packed, with heights in the range 300-600 nm, and a lateral, spatial frequency in the range 5–7 μm^−1^. A reflectance in the visible spectrum as low as 6%, and an absorbance of 90% was observed for an Al film of 100 nm thickness. Corresponding experiments on a planar film yielded 80% reflectance and 20% absorbance. The observed absorbance enhancement is attributed to a gradient effect causing the metal film to be antireflective, analogous to the mechanism in dielectrics and semiconductors. We find that the investigated nanostructures have too large spatial frequency to facilitate efficient coupling to the otherwise non-radiating surface plasmons. Applications for decoration and displays are discussed.

Despite being highly absorbing, the optical appearance of metals are characterized by a very high reflectance. More than 90% of the light is typically reflected at the interface of the metal, and is never allowed to enter the bulk, to eventually be absorbed. This interface however, can be altered using nanostructures which can lower the reflectivity of the metal surface, allowing the light to enter the bulk. A metal surface can thus be changed from being highly reflective, to appearing completely black, simply by structuring the surface.

Using nanostructures to lower the reflectivity of dielectric and semiconducting materials has been known for many years, the so-called moth-eye structures^1^,^2^,^3^,^4^,^5^. But only in recent years have the optical properties of nanostructured metal surfaces been investigated.

Vorobyev *et al.* have studied blackening of various metals by femtosecond laser ablation^6^. The metal surfaces form micro and nanostructures during the ablation, and Vorobyev *et al.* suggest broad surface-plasmon resonances in the nanostructures, as well as light trapping from the microstructures as the cause of the increased absorbance.

Søndergaard *et al.* reported a 96% absorbance in 900 nm thick gold films, using adiabatic nanofocusing of gap surface plasmons in ultrasharp ion beam milled grooves^7^. Raza *et al.* later showed that asymmetry in the fabricated nanostructures play a crucial role in the high absorption of such nanostructures^8^.

Thin metal films on moth eye structures have also been suggested for use in heat-insulating filters. Here the nanostructures increase the transmittance of the filters in the visible spectrum^9^,^10^.

Other approaches have been investigated, relying on thin-film interference for lowering the reflectance. Sang-Hwan *et al.* fabricated a 4-layer dielectric-metal-dielectric-metal structure, which showed 99.3% average absorbance in the visible spectrum^11^. Jen *et al.* fabricated metal-dielectric-metal pillars on a glass substrate with an absorbance of 80% and 40% for light with a wavelength of 400 nm and 800 nm, respectively^12^.

Studies on resonant grating-waveguide structures further show how gratings can be used to modify the optical properties of a surface. Such structures typically result in highly resonant phenomena^13^,^14^,^15^,^16^.

Absorbing metal films could potentially be used in photovoltaics, and thermo-photovoltaics, as well as for increasing the contrast of flat-panel displays. Or even for their visual characteristics, similar to coloured anodized aluminium oxide. Metal deposited on periodic pillar arrays can even be designed for reflecting specific colours^17^,^18^.

Here we report an approach to increase the absorption of metals, by depositing thin metal films on a dielectric substrate with imprinted antireflective black silicon (BSi) moth-eye nanostructures. We thus reduce the reflectance of Al films from 80% to 6%, and increase the absorbance from 20% to over 90%, in a 100 nm thick film.

## Results

### Characterization and visual appearance

**Figure 1:**
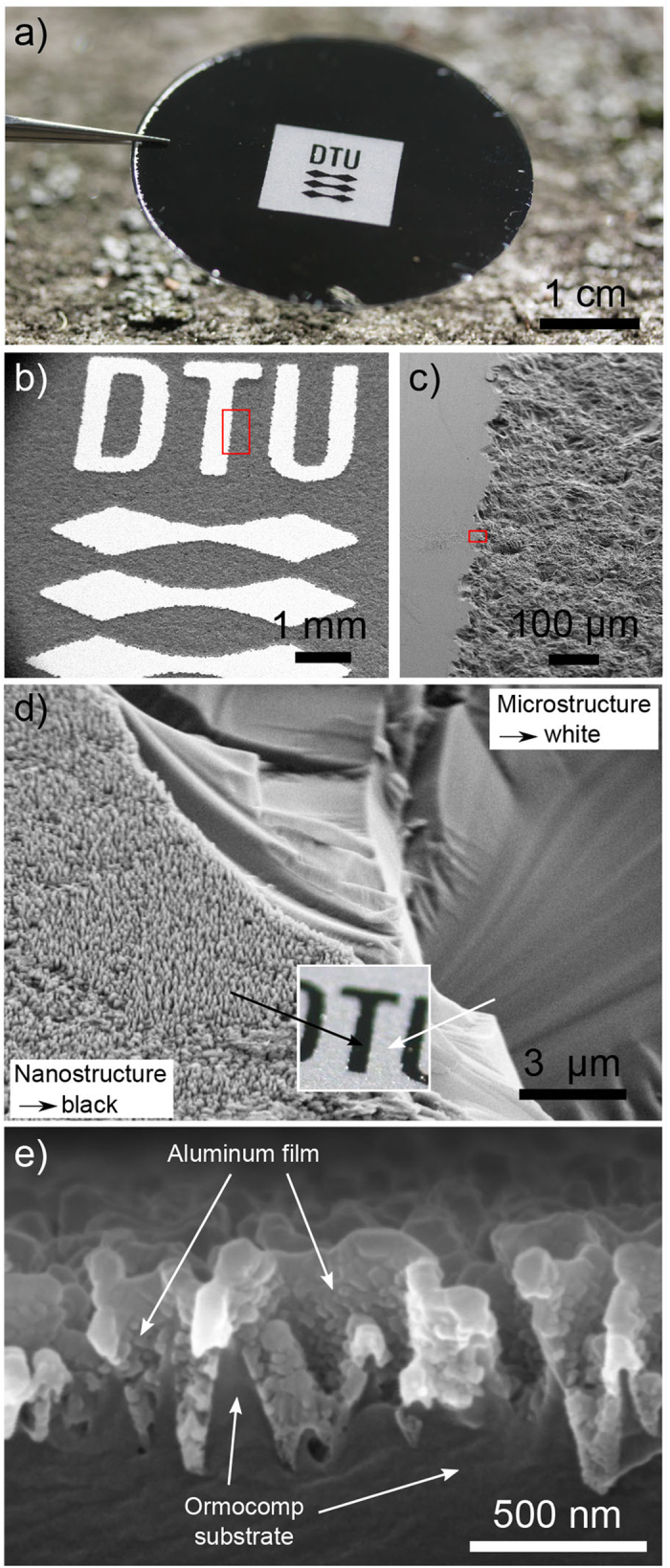
Photograph of black metal film and SEM visualization of surface structures. (**a**) Photograph of a sample consisting of a 100 nm Al film deposited on a nanostructured Ormocomp substrate. On the black areas, the Ormocomp is nanostructured, while on the bright logo, the Ormocomp has a rough surface, causing a diffuse reflecting metal surface. (**b**–**d**) Scanning electron micrographs of the nano- and microstructured Si master, leading to the black and white logo shown in (**a**). (**e**) Cross section of Ormocomp sample (type C) with 100 nm Al.

Figure 1a showcases the black metal film when deposited on a nanostructured Ormocomp (Micro resist technology Gmbh, Berlin) substrate. The grey area forming the logo, is where the Ormocomp is rough but not nanostructured, resulting in a diffuse reflecting surface. The different structure sizes of the roughness and the nanostructured surfaces is displayed in Fig. 1b–d, showing SEM images of the Si master. Figure 1e shows an SEM cross section of an Ormocomp substrate (type C) with a 100 nm thick Al film.

**Figure 2:**
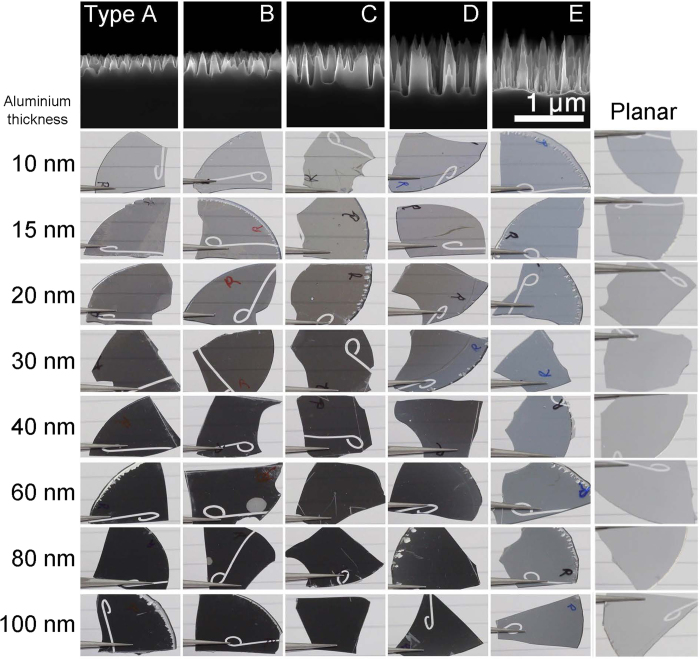
Photographs of thin Al films on nanostructured Ormocomp samples. The type of BSi structure is shown on side-view scanning electron micrographs in the top row. Photos are taken from the Ormocomp-metal interface. Each column shows structures of type A to E, as well as a planar Ormocomp sample. Each row represents an Al film thickness from 10 to 100 nm.

Different types of BSi nanostructures where studied. SEM images of the structures are shown in the upper row in Fig. 2. The geometric parameters of the different structures were measured from SEM images, and are summarized in Table 1. The spatial frequency was calculated using Fourier transformation of top-view SEM images. It has previously been shown that when replicated into a transparent, dielectric material, these nanostructures exhibit antireflective properties^19^.

The replicated Ormocomp samples are shown in Fig. 2, after deposition of Al thin films with thickness varying from 10-100 nm. The samples were photographed when held in a distance of 2 cm from the surface, allowing for the lines in the paper to be seen on samples with a semi-transparent film. For the structures of type A-D, the metal films became more opaque with increasing film thickness, but retained a low reflectance while appearing black. The planar reference became opaque, with a mirror-like appearance, as expected. The type E structures appeared grey.

### Optical measurements

**Figure 3:**
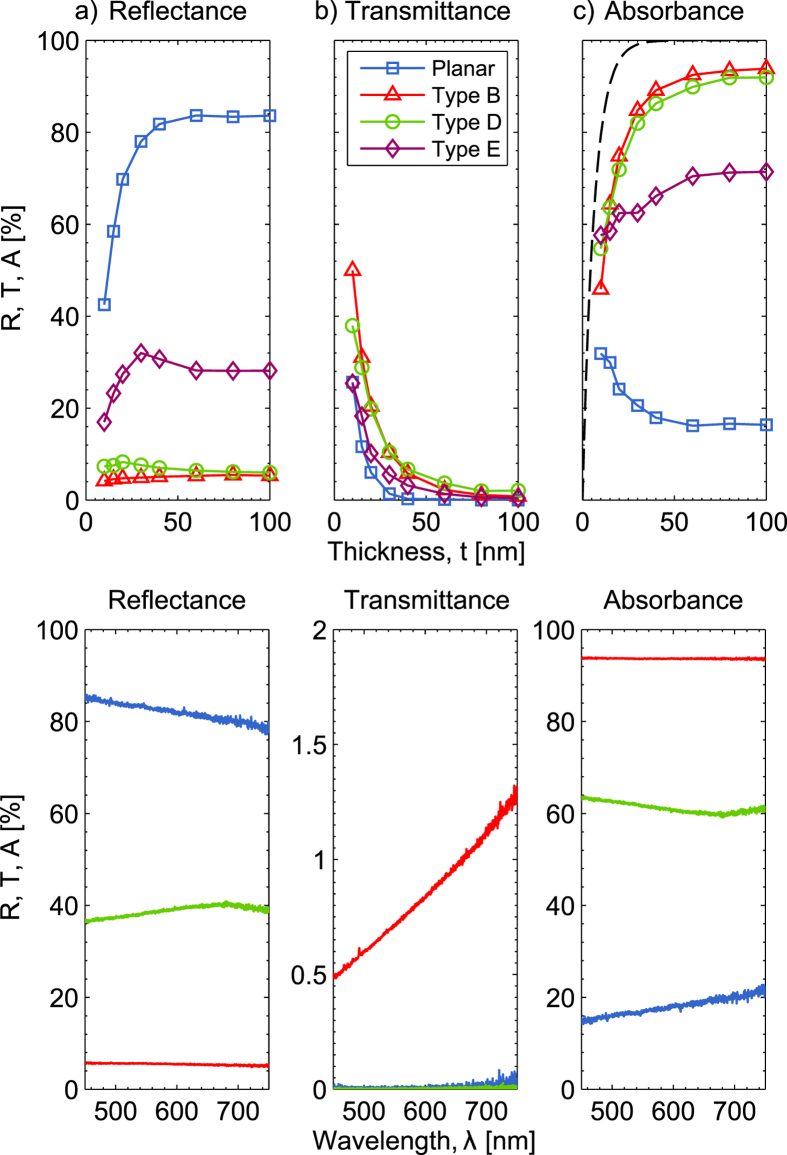
Optical measurements. (**a**) Total reflectance measured with integrating sphere, as function of metal thickness. (**b**) Specular transmittance at 500 nm wavelength, versus Al film thickness. (**c**) Absorbance at 500 nm wavelength, versus Al film thickness. The dashed line shows the decay rate of an electromagnetic wave traveling in bulk Al. Absorbance was measured as A = 1 − *R*_total_ − *T*_specular_. (**d**–**f**) Spectral reflectance, transmittance and absorbance for type B structure, planar and powder blasted, rough structure.

Figures 3a–c show the measured reflectance, transmittance and absorbance as function of deposited Al thickness, for light with 500 nm wavelength, for a planar sample, and nanostructured samples of type B, D, and E. The reflectance of the planar sample increases rapidly with the increasing Al film thickness, 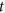, to around *R* = 80%. This is due to the short skin depth of Al, for which the theoretical value at 500 nm wavelength is 6.4 nm, ensuing that the film is optically dense for thicknesses above around 40 nm. The type B structures have significantly lower reflectance at *t* = 10 nm, and only show a slight increase, when Al thickness is increased to *t* = 100 nm, up to around *R* = 6%.

The transmittance of the samples is plotted in Fig. 3b, showing that for all samples, the transmittance decreases rapidly with increasing metal thickness, as would be expected. The decrease in transmittance is slower for the nanostructured films though, and the nanostructured thin films retain a transmittance of around T = 1%, at *t* = 100 nm.

The absorbance of the planar Al film is roughly 30% at 10 nm thickness, and decreases to roughly 20% at 60-100 nm thickness. The absorbance of the Al films deposited on nanostructures of type B and D show a dramatic increase in absorbance, when increasing the Al thickness. For a thickness of *t* = 100 nm, the absorbance is above 90% for the type B structures. This is mainly due to the very low reflectance of the films. Note that the absorption properties of the nanostructured films approach the decay rate of an electromagnetic wave travelling in bulk Al, which is given by the Beer-Lambert law, and is shown as a dashed line. The results for type A and C structures are not shown as they are very similar to those of type B.

Figures 3d–f show the spectral optical properties of a 100 nm thick Al film on a planar sample, a type B Ormocomp sample, and on the rough, powder blasted surface shown in Fig. 1a. The optical response from all three samples show very low dispersion in the optical spectrum. In particular, the type B nanostructures result in very low dispersion in the reflectance and absorbance, which give rise to the black appearance of the metal films. The reflectance of the metal film on a rough surface is significantly lower than a planar film, at a round 40%. However, this is not enough to render the surface black, as seen if Fig. 1a.

The results suggest that the nanostructures should be tapered and closely spaced to effectively suppress reflections. For the nanostructures investigated here, structures with a height in the range 300-600 nm showed very similar results. The thickness of the nanostructured Al film should be 100 nm, to obtain an almost optically dense film.

The possible role of plasmon excitations? The structured surface could suggest that coupling to surface plasmon polaritons (SPPs) could occur, analogous to grating coupling. The theory of grating coupling explains how the grating may contribute the additional momentum *G* required for light to couple from the unbound modes in the polymer to the bound SPP modes at the interface^20^, i.e. *k*(*ω*) *+* *G* *~* *β*(*ω*) with *k*(*ω*) = *ω* /(*nc*) being the free-space wave number while *β*(*ω*) is the wave number of the surface plasmon. Here, we may imagine that the rough surface acts as randomized grating with a characteristic distribution of grating periods^19^.

**Figure 4:**
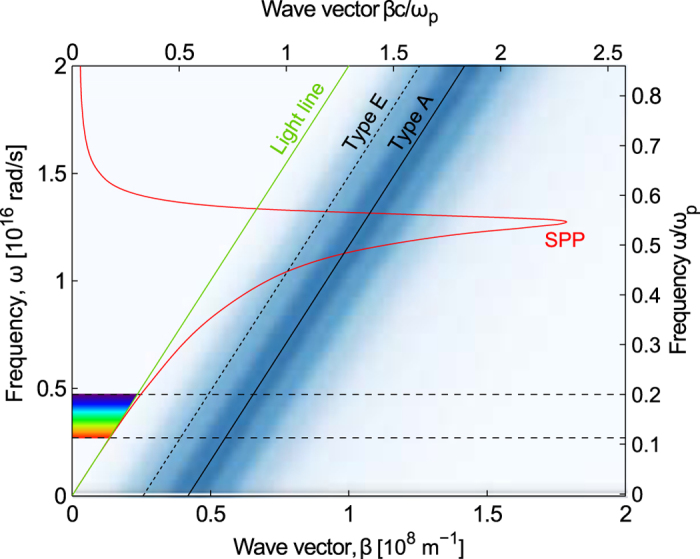
Dispersion relation for surface plasmon polaritons Dispersion relation for SPP on the interface between a semi-infinite medium of polymer (refractive index *n* = 1.5), and Al. The shaded blue area represents the momentum distribution contributed by type A surface structures, as calculated using Fourier methods from SEM images of the nanostructured surfaces. The solid line represents the maximum of the momentum distribution of the type A nanostructures, while the dashed line represents the maximum for the type E nanostructures. It is evident that in the visible spectrum, only a very small overlap exists between the SPP modes in the film, and the momentum distribution of the nanostructured surface.

Figure 4 shows the dispersion relation for SPP on the interface between semi-infinite media of polymer, and Al (Al is modelled using the complex refractive index by Rakić^21^). The shaded blue area represents the distribution of the momentum contributed by the surface structures, as calculated from Fourier methods from SEM images of the nanostructured surfaces. From the Fourier analysis, a power spectrum is calculated, which represent the spatial frequencies of the structures in the surface. The added momentum *G* from these spatial frequencies *p* are then calculated as G = 2π*p* , and offset in the plot by the light line.

Within the visible spectrum there is no significant overlap between the SPP modes and the momentum added by the surface structures, while coupling appear feasible at only higher frequencies (*ω* ~ 1.1 × 10^16^ *s*^−1^). The characteristic spatial frequencies of the nanostructures are too large to contribute to significant coupling in the visible spectrum.

The low dispersion observed in the reflectance and transmittance spectra in Fig. 3d–e, further suggests that there is no resonant behaviour, which is otherwise commonly observed in the plasmonic response of nanostructured thin films^22^.

We further note that the structures cause an increase in the light transmittance through the thin film. This could be caused by a decrease in the effective film thickness due to the nanostructured surface. The increased transmittance again suggests that there is no additional cause of absorbance in the film.

Finally, a type A Ormocomp substrate with a 20 nm Al film was investigated using near-field scattering optical microscopy (NSOM), showing SPP excitations in the film (See Fig. S1 in supplementary material). The aperture of the NSOM probe allows for coupling to the SPP modes in the film (See Fig. S2 in supplementary material).

Other materials have also been investigated, and despite having very different or no plasmonic properties, nanostructured thin films of Au, Cr, and Ge, all behave similar to Al (See Fig. S3 and S4 in supplementary material).

In conclusion on these observations, we have found no evidence that coupling to plasmons in the metal film of metal particles on the surface contribute to the absorbance of light in the nanostructured metal films, for free space light.

We therefore suggest that the increased absorbance is mainly due to an antireflective gradient effect, similar to the moth-eye effect. Instead of being reflected, the light is allowed to be transmitted in to the metal film, where it is absorbed. The mechanism of the enhanced absorbance is thus similar in nature, to that of a semiconductor, e.g. in black silicon.

### Applications

**Figure 5:**
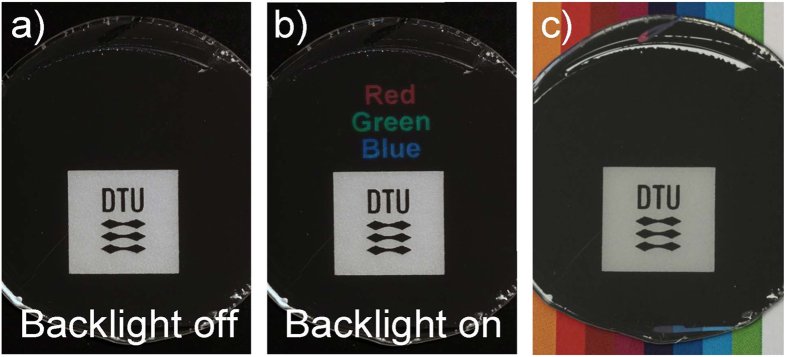
Photographs of black metal films used for displays or decorations. (**a**) Back light off: the light display is hidden. (**b**) Back light on: the light display is seen as light is transmitted through the black metal film. (**c**) Without backlighting, colourful backgrounds are not visible through the film, due to the limited transmittance.

Figure 5 shows the sample seen in Fig. 1a, with different backgrounds. Figure 5a–b shows the sample in front of a backlit display when the back light is off (Fig. 5a), and when the display is on (Fig. 5b). If placed directly on a colourful surface, the background is on the other hand not visible, due to the low transmittance of the metal film (Fig. 5c). We thus showcase how the nanostructured metal films e.g. could be used in design elements. The nanostructured surface is protected by a transparent coating, and is therefore abrasion resistant.

The advantage of the fabrication approach presented here is that the substrate can in principle be any material, and can thus potentially be manufactured by industrial methods like injection moulding or roll-to-roll imprinting, followed by metal deposition. The fabrication is similar to recent studies on plasmonic structural colour effects^18^, suggesting that the two methods could be combined to form vivid colours, contrasted by the deep black films presented here.

The very small skin depth of e.g. Al means that almost all the incoming light can be absorbed in films with thickness of few tens of nanometres. Very small amounts of Al are thus needed to form the strong optical effects, suggesting that metal coated polymers could be reused with only very small contamination from the metal film.

## Discussion

We have fabricated and explored highly absorptive metal thin films with low reflectance, using low cost black silicon masters. By depositing thin metal films on a nanostructured Ormocomp substrate, the reflectance of the Ormocomp-metal interface was decreased from 80% for a planar metal film, to 6% for a nanostructured film. The absorbance was increased from 20% to over 90%, for 100 nm Al films, when illuminated from the Ormocomp-metal interface. The transmissivity of the nanostructured films increases significantly, compared to a planar film with no nanoscale roughness.

The results suggest that the nanostructures should be tapered and closely spaced to effectively suppress reflections. For the nanostructures investigated here, structures with a height in the range 300-600 nm, and a lateral, spatial frequency in the range 5–7 μm^−1^ showed very similar results. A nanostructured Al film with a thickness of 100 nm resulted in an almost optically dense film.

Finally, we find no evidence of plasmonic excitations playing a crucial role in the observed increase in absorbance of the films. We therefore suggest that the observed effect is mainly due to an antireflective effect, causing a dramatic decrease in the reflectance of the films, thereby allowing the light to be absorbed in the thin metal films.

## Methods

### Fabrication

The BSi substrates were structured by reactive-ion etching (Pegasus DRIE, STS). The structures were formed in a single etching cycle with an O_2_/SF_6_-based etch. Different structures were obtained by varying the O_2_:SF_6_ flow ratio^23^. The parameters are summarized in Table 2. The grey area forming the logo on the sample shown in Fig. 1a was obtained by powder blasting the Si master, resulting in removal of the nanostructures, and formation of a rough surface. The BSi masters were coated with an anti-stiction layer (perfluorodecyltrichlorosilane) using molecular vapour deposition. The masters were then used in a UV-NIL process for replicating the structures into Ormocomp, a transparent organic-inorganic hybrid polymer (Micro resist technology Gmbh, Berlin). Ormocomp was poured on the BSi master, and a PMMA substrate was applied, to level the Ormocomp surface. The Ormocomp was exposed to UV light for 5 minutes by illumination through the PMMA. After the exposure, the Ormocomp was released from the BSi master, and the PMMA. See Christiansen *et al.*^19^ for details. Thin metal films were deposited on the structured Ormocomp samples, using electron beam physical vapour deposition (Alcatel SCM 600), at a rate of 15 Å/s. The thickness of the metal layer was controlled by a piezo-electric crystal in the evaporation chamber.

### Optical Measurements

The total and diffuse reflectance was measured using an integrating sphere (Ocean Optics ISP50-8-R-GT) with a diameter of 50 mm. The incoming white light is incident in an angle of 8 degrees from normal incidence. As reference for the reflectance measurements we used a 100 nm thick Al film on a borofloat glass wafer. For the specular transmission measurements, the samples were illuminated at normal incidence with white light (Xenon lamp, HPX-2000, Ocean optics), through a fiber and a collimator. The samples were aligned perpendicular to the incident light using a goniometer. The light was then collected in a fiber with a collimator, and analyzed in a spectrometer (Jaz, Ocean Optics). The absorbance of the metal films was calculated as A = 1 − *R*_total_ − *T*_specular_.

## Additional Information

**How to cite this article**: Christiansen, A. B. *et al.* Black metal thin films by deposition on dielectric antireflective moth-eye nanostructures. *Sci. Rep.* **5**, 10563; doi: 10.1038/srep10563 (2015).

## Supplementary Material

Supplementary Information

## Figures and Tables

**Table 1:** Measured height and spatial frequency of the fabricated BSi masters.

**Type**	**Height [nm]**	**Spatial frequency,** 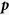 **[μm^−1^]**
A	315±35	6.7±0.5
B	450±50	6.3±0.4
C	615±80	5.1±0.3
D	815±120	4.3±0.3
E	880±140	4.1±0.3

**Table 2:** Reactive-ion etching parameters for the BSi masters. Only the oxygen flow was varied, with increments of 10 sccm.

	**Structure type**				
**Parameters**	**A**	**B**	**C**	**D**	**E**
O_2_ flow [sccm]	110	100	90	80	70
SF_6_ flow [sccm]			70		
Etch time [min]			8		
Platen power [W]			30		
Coil power [W]			2700		
Chamber pressure [mTorr]			6		
Temperature [°C]			−10		
