# Supplementary material for: Black metal thin films by deposition on dielectric antireflective moth-eye nanostructures
Source: Sci Rep. 2015 Jun 2;5:10563. doi: 10.1038/srep10563 (PMC4649914; doi:10.1038/srep10563)
Supplement: Supplementary Information [file srep10563-s1.pdf]

Black metal thin films by deposition on dielectric antireflective moth-eye nanostructures: Supplementary material

Alexander B. Christiansen,[1] Gideon P. Caringal,[1] Jeppe S. Clausen,[2] Meir Grajower,[3] Hesham Taha,[4] Uriel Levy,[3] N. Asger Mortensen,[2] and Anders Kristensen[1, a)]

[1)] *Department of Micro and Nanotechnology, Technical University of Denmark, Oersteds Plads, Building 345B, DK-2800 Kgs. Lyngby, Denmark.*

[2)] *Department of Photonics Engineering, Technical University of Denmark, Oersteds Plads, Building 343, DK-2800 Kgs. Lyngby, Denmark.*

[3)] *Department of Applied Physics, The Benin School of Engineering and Computer Science, The Center for Nanoscience and Nanotechnology, The Hebrew University of Jerusalem, Jerusalem 91904, Israel*

[4)] *Nanonics Imaging Ltd., Hartum 19, Har Hotzvim, Jerusalem 97775, Israel*

[a)] Electronic mail: anders.kristensen@nanotech.dtu.dk

I. NEAR-FIELD SCANNING OPTICAL MICROSCOPY MEASUREMENTS

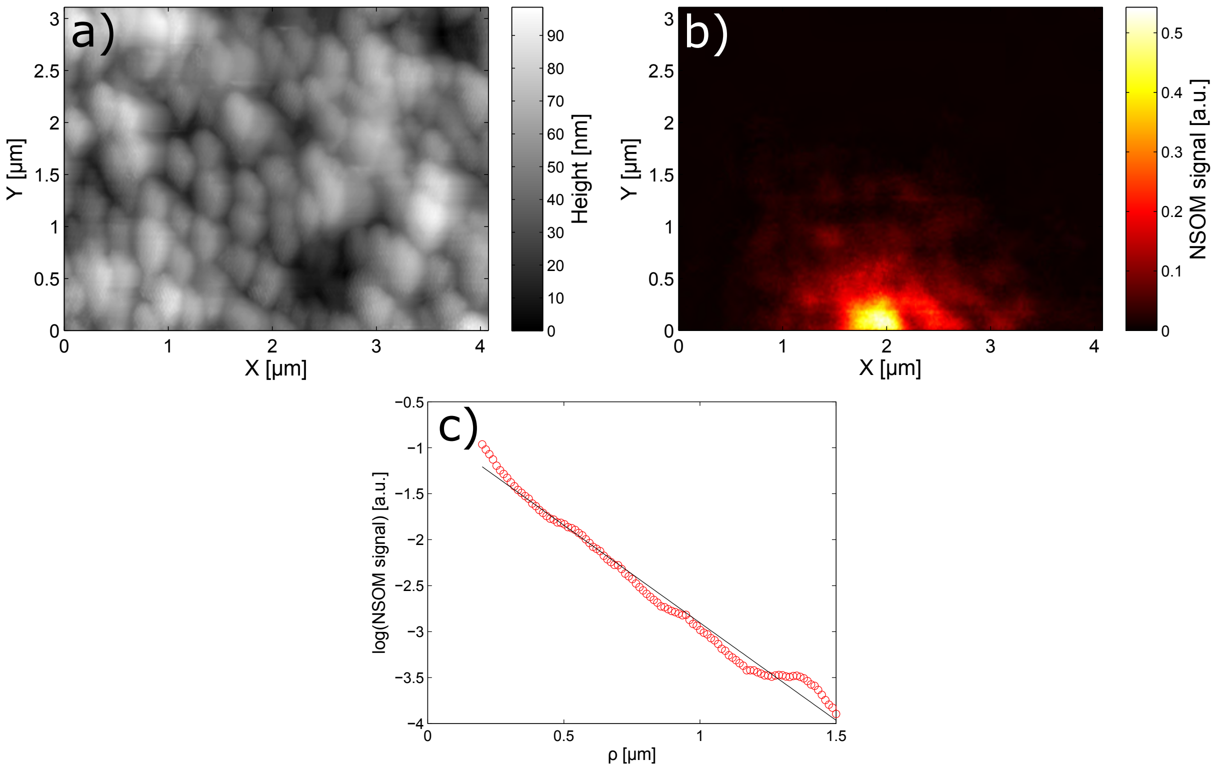

FIG. S1. **Near-field scanning optical microscopy (NSOM) measurements.** The near-field measurements were carried out using a dual probe near-field scanning optical microscope (NSOM, MultiView4000TM, Nanonics Imaging Ltd.), where the excitation was done using one tapered metal coated fiber probe at a fixed position, and the near field was measured with another probe by scanning an area in the close vicinity of the excitation. a) topographic AFM data of type A Ormocomp surface with a 20 nm thick Al film, recorded simultaneously with the NSOM signal. b) NSOM signal from surface in (a). The excitation occurred just below the bottom center of the image. c) Plot of NSOM signal after azimuthal averaging around the excitation probe. The observed modes show a propagation length of roughly 500 nm, where the signal has decayed to a factor of 1/e of the initial value.

The Near-field scanning optical microscopy (NSOM) measurements were made using an Optometronic 4000 dual probe NSOM system[1]. The NSOM system was an aper-

ture operation system, using apertures for both excitation and collection. Both probes were metal coated optical fibers that were placed in close contact to the surface. The excitation fiber had a 100 nm aperture, and the collector an aperture of 200 nm, i.e. both probes being significantly larger than the characteristic length scales of the surface roughness (see Table I in the main text). A laser with a wavelength of 532 nm was used for the measurement. During operation, the exciting fiber was held in a fixed position on the surface, while the collecting fiber was scanned over the surface. Both probes were controlled in tapping mode (shear force feedback mode). The collecting probe works as a standard AFM probe, as well as an NSOM probe. Thus, two images are recorded at the same time; topographic data and the NSOM signal.

The observed modes show a propagation length of roughly 500 nm, where the signal has decayed to a factor of 1/e of the initial value. The theoretical propagation length of an infinite, planar Al film can be calculated as discussed in the book by Maier, in chapter 2.3[2]. For a 20 nm thick film, SPPs on either face of the film can couple, which allows for the existence of two modes: an even and an odd mode. For a 20 nm Al film surrounded by a medium with refractive index $n = 1$ on one side, and $n = 1.5$ on the other side, the SPPs will have propagation lengths of 2.1 µm and 11.2 µm for the even (symmetric in the electric field), and odd mode respectively.

Figure S2 shows the dispersion relation for the even and odd mode for SPPs on a 20 nm thin Al film between two semi-infinite media with refractive index, $n = 1.5$. For the thin film, the dispersion splits up in two modes, as discussed by Maier, chapter 2.3[2].

The shaded blue area represents the momentum distribution contributed by the 100 nm diameter circular aperture in the exciting NSOM probe. For a thin film, the momentum contributed by the NSOM probe allows coupling to both the even and odd mode, for light with 532 nm wavelength (the middle dashed line).

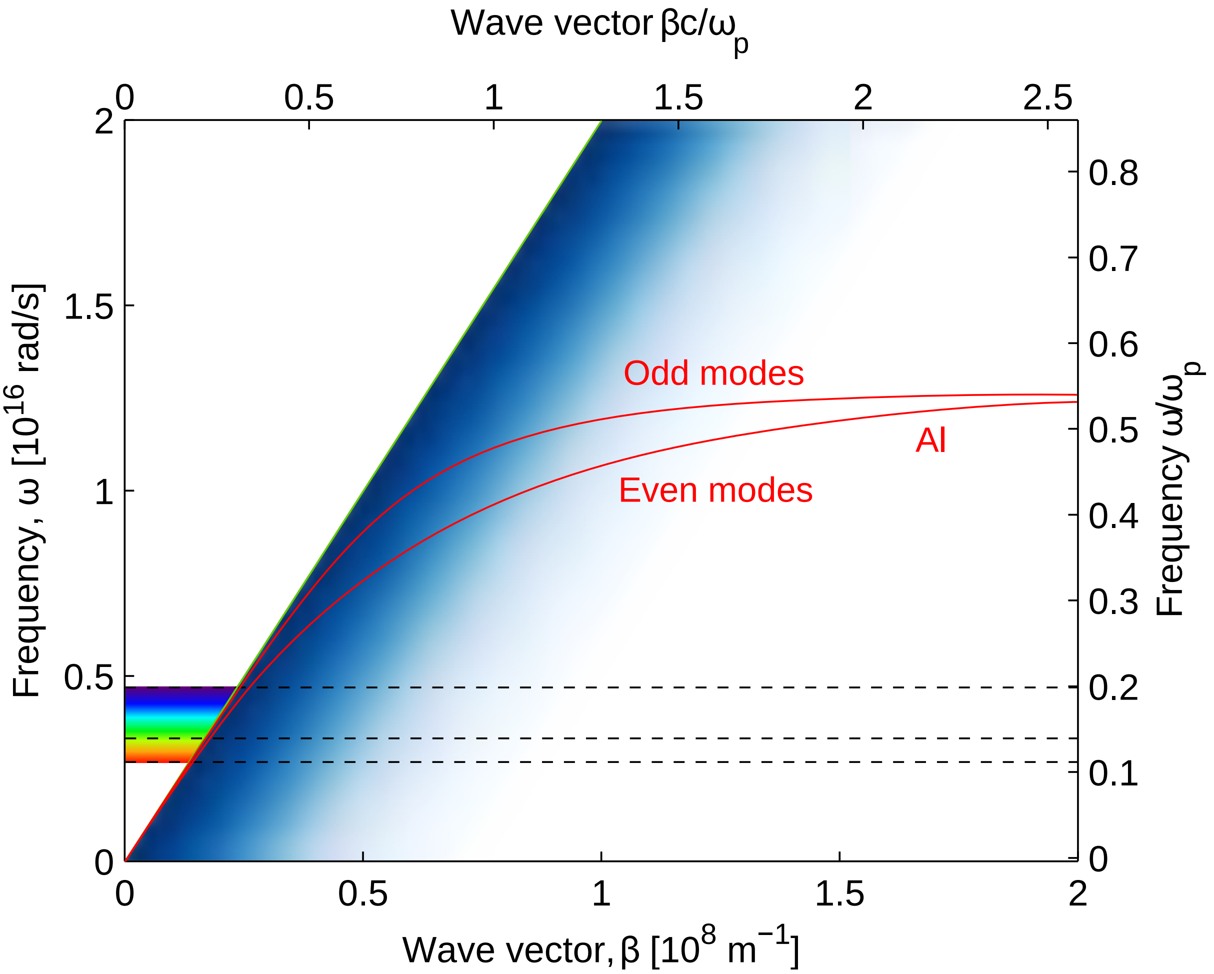

FIG. S2. **NSOM coupling to plasmons** Dispersion relation for SPPs on a 20 nm thin Al film between air (n=1) and a polymer (n=1.5). The shaded blue area represents the momentum distribution contributed by the 100 nm diameter circular aperture in the exciting NSOM probe. The middle dashed line represents the 532 nm wavelength of the exciting laser. In the near field the probe thus allows fro coupling to SPP modes on the Al film.

II. NANOSTRUCTURED AU, CR, AND GE FILMS

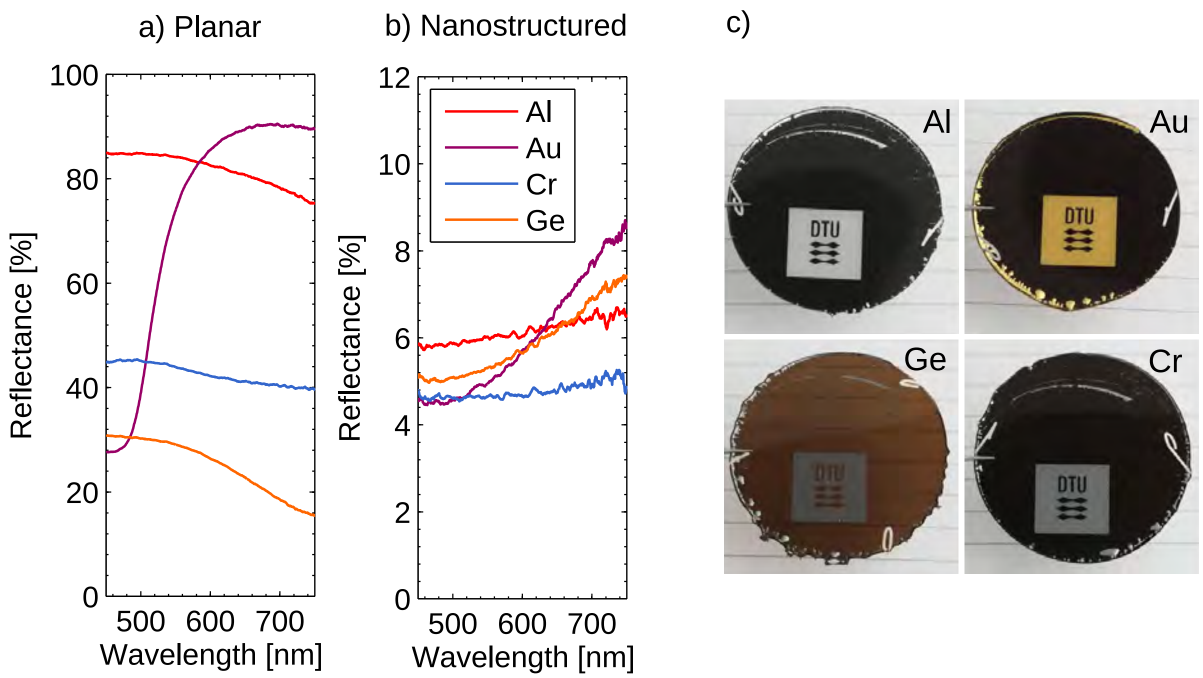

FIG. S3. **Reflectance spectra and photos of planar and nanostructured metals.** Total reflectance of planar films (a) and type B nanostructured films (b). The reflectance was measured from the Ormocomp-metal interface. (c) Photos of Ormocomp samples with Al, Au, Ag, Cr, and Ge deposited. The logo on each sample is where the Si master was powder blasted before casting the Ormocomp. The surface here is not nanostructured, but rather microstructured.

Nanostructured thin films of Au, Cr, and Ge were also investigated. Au and Cr were deposited using electron-beam evaporation, while Ge was deposited using thermal deposition (Wordentec QCL 800 system). The films were deposited on a type B Ormocomp substrate, and the optical properties of the metal-Ormocomp interface were characterized. Figure S3(a) shows the reflectance of planar films deposited on a glass substrate, while Fig. S3(b) shows the reflectance of the nanostructured films. For all materials, the nanostructures significantly decreases the reflectance of the films, resulting in black films for Au and Cr, while for Ge, the absorbance is too low so that the film is still semi-transparent with a brown tint.

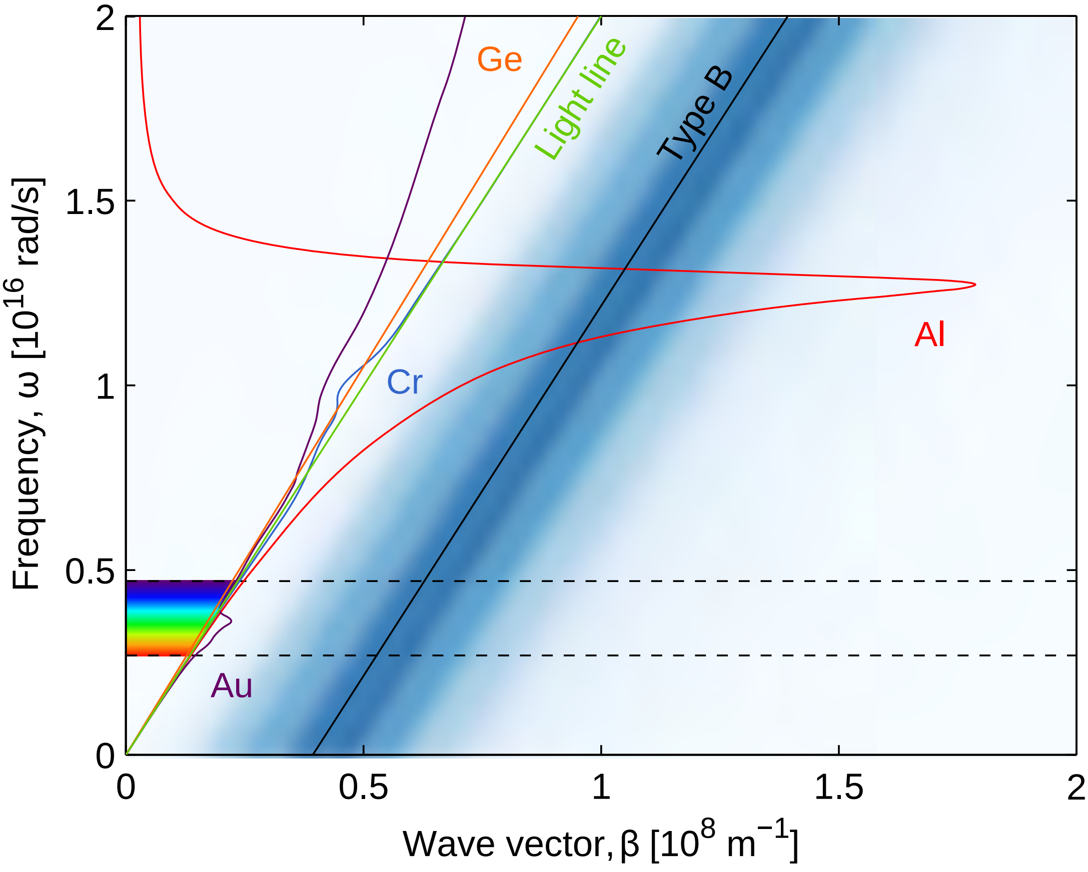

FIG. S4. **Dispersion relation for surface plasmon polaritons for different materials.** Dispersion relation for SPPs on the interface between a semi-infinite medium of polymer (refractive index $n = 1.5$), and Al, Au, Cr, and Ge. The shaded blue area represents the momentum distribution contributed by type B surface structures, as calculated using Fourier methods from SEM images of the nanostructured surfaces. The solid line represents the maximum of the momentum distribution of the type B nanostructures.

Figure S4 shows the dispersion for SPPs on the interface between a semi-infinite medium of polymer (refractive index $n = 1.5$), and Al, Au, Cr, and Ge (refractive index data from Rakic *et al.*[3] and Icenogle *et al.*[4], data available from http://refractiveindex.info[5]). Despite the very different SPP dispersion relations, the different materials behave very similarly when deposited on the nanostructured substrates, in that they all show a dramatic decrease in reflectance, as shown in Fig. S3.

REFERENCES

[1] "Nanonics Imaging Ltd., Jerusalem, Israel,".

[2] S. A. Maier, *Plasmonics: fundamentals and applications* (Springer, 2007).

[3] A. Rakic, A. Djurišic, J. Elazar, and M. Majewski, Applied optics **37**, 5271 (1998).

[4] H. W. Icenogle, B. C. Platt, and W. L. Wolfe, Applied optics **15**, 2348 (1976).

[5] M. N. Polyanskiy, "Refractive index database,".
